# The human odorant receptor OR10A6 is tuned to the pheromone of the commensal fruit fly *Drosophila melanogaster*

**DOI:** 10.1016/j.isci.2022.105269

**Published:** 2022-10-03

**Authors:** Tim Frey, Charles A. Kwadha, Franziska Haag, Julien Pelletier, Erika A. Wallin, Elsa Holgersson, Erik Hedenström, Björn Bohman, Marie Bengtsson, Paul G. Becher, Dietmar Krautwurst, Peter Witzgall

**Affiliations:** 1 Leibniz-Institut für Lebensmittel-Systembiologie an der Technischen Universität München, Lise-Meitner Strasse 34, 85354 Freising, Germany; 2 Department of Plant Protection Biology, Swedish University of Agricultural Sciences, Box 190, 234 22 Lomma, Sweden; 3 Department of Chemical Engineering, Mid Sweden University, Holmgatan 10, 85170 Sundsvall, Sweden; 4 Systembolaget AB, 103 84 Stockholm, Sweden

**Keywords:** Biological sciences, Biochemistry, Sensory neuroscience

## Abstract

All living things speak chemistry. The challenge is to reveal the vocabulary, the odorants that enable communication across phylogenies and to translate them to physiological, behavioral, and ecological function. Olfactory receptors (ORs) interface animals with airborne odorants. Expression in heterologous cells makes it possible to interrogate single ORs and to identify cognate ligands. The cosmopolitan, anthropophilic strain of the vinegar fly *Drosophila melanogaster* depends on human resources and housing for survival. Curiously, humans sense the pheromone (*Z*)-4-undecenal (Z4-11Al) released by single fly females. A screening of all human ORs shows that the most highly expressed OR10A6 is tuned to Z4-11Al. Females of an ancestral African fly strain release a blend of Z4-11Al and Z4-9Al that produces a different aroma, which is how we distinguish these fly strains by nose. That flies and humans sense Z4-11Al via dedicated ORs shows how convergent evolution shapes communication channels between vertebrate and invertebrate animals.

## Highlights

• Humans sense the sex pheromone Z411-Al released by single *Drosophila melanogaster* females
• The most highly expressed human olfactory receptor OR10A6 is tuned to Z411-Al
• An African fly strain emits two aldehydes, which we distinguish from Z411-Al by nose
• Convergent evolution shapes chemical communication between phylogenies

## Graphical abstract

**Figure:**
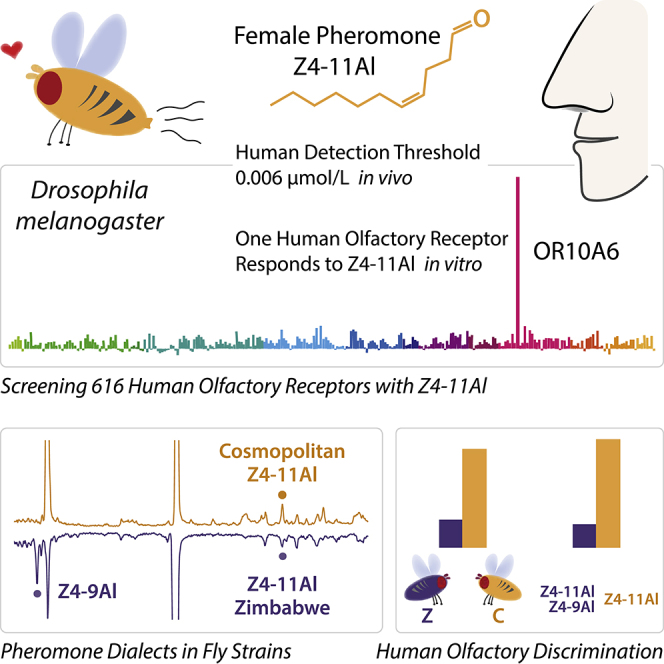

## Introduction

Volatiles from animals, microorganisms, and plants are yielded by basic metabolic pathways, they inform about resources and habitats, and reveal identities and social context. Communication via volatile chemicals is reliable and inclusive, for all living beings across the kingdoms.

Volatiles are aired tweets for those equipped with receptors and sensory circuits to capture and interpret them. Animals possess olfactory receptors (ORs) for peripheral detection of volatiles, and for filtering out scents that make ecological and behavioral sense, from a noisy chemical airspace. Functional evolution and adaptation of ORs to ecosystem and habitat cues and to social and sexual signals reflects their importance in interfacing animals with the chemical environment (Hayden et al., 2010; Fleischer et al., 2018; Robertson, 2019; Saraiva et al., 2019; Auer et al., 2020; Marcinek et al., 2021; Prieto-Godino et al., 2021). Olfaction has developed independently in invertebrates and vertebrates, but the overarching organization and functional logic of the olfactory system, building on rapidly evolving ORs, expressed in peripheral olfactory sensory neurons, feeding into a hierarchy of central olfactory circuits, is convergent (Ache and Young, 2005; Su et al., 2009; Bear et al., 2016; Wang et al., 2021).

A principal, current objective, and fascinating challenge in vertebrate and invertebrate olfaction research is to explore the receptive range of ORs and to set landmarks in chemical space, for comprehension of olfactory codes and the functional analysis of olfactory systems. Expression of ORs in heterologous cell systems, for example in human embryonic kidney (HEK) cells, enables experimental access and makes it possible to interrogate individual ORs and to identify their cognate ligands (Krautwurst et al., 1998; Corcoran et al., 2014). Human ORs are seven-transmembrane domain G-protein coupled receptors and the first challenge is to achieve fully functional membrane expression (Noe et al., 2017b). The ensuing step is to compose comprehensive and manageable, yet representative panels of biologically relevant compounds for investigating their receptive range. One strategy is to use key food odorants, identified from food and beverages (Krautwurst and Kotthoff, 2013; Dunkel et al., 2014). Odorant panels will, however, always remain notoriously incomplete – in comparison with an overwhelmingly diverse odorscape, containing countless chemicals. *In vitro* OR screenings do afford active ligands, but most human ORs remain in the orphan state (de March et al., 2015; Block, 2018; Haag and Krautwurst, 2021).

Chemicals we perceive in very small amounts, and which are not strictly associated with food, are inspiring targets for OR screenings. One such candidate compound is (*Z*)-4-undecenal (Z4-11Al), the volatile female pheromone of the commensal fruit fly *D. melanogaster*. In *Drosophila*, two isoforms of DmelOR69a (Robertson et al., 2003), with dual specificity for food odorants and pheromone, are co-expressed in the same OSNs. Intriguingly, we ourselves readily perceive Z4-11Al, which is released at subnanogram amounts per hour. As only females produce this scent, we reliably distinguish between male and female flies (Lebreton et al., 2017; Becher et al., 2018).

Cosmopolitan *D*. *melanogaster* flies are strictly anthropophilic. They accompanied the human expansion from out of Africa around 10.000 ya, and have been isolated from African flies, such as the Zimbabwe strain (Lachaise and Silvain, 2004; Arguello et al., 2019; Sprengelmeyer et al., 2020). The cosmopolitan fly pheromone Z4-11Al is an oxidation product of the aphrodisiac cuticular hydrocarbon (*Z*,*Z*)-7,11-heptacosadiene (Z7,Z11-27Hy) (Billeter et al., 2009; Lebreton et al., 2017). Because Zimbabwe females produce more (*Z*,*Z*)-5,9-heptacosadiene (Z5,Z9-27Hy) than Z7,Z11-27Hy (Dallerac et al., 2000; Grillet et al., 2012), consequently these flies would release another aldehyde. Posing that our perception of Z4-11Al is not only sensitive but also specific, we asked whether we are able to olfactorily discriminate between females of the cosmopolitan and Zimbabwe strains of *D*. *melanogaster*. A sensory panel corroborated this idea by comparing synthetic compounds and fly odors.

Naturally, this invites the question – how do humans smell the scent of the fly? A range of human ORs is tuned to straight-chain aldehydes, which are commonly found in fruit and vegetable aromas (Schmiedeberg et al., 2007; Saraiva et al., 2019; Nara et al., 2011; Li et al., 2014; de March et al., 2015; Block 2018) and perception of Z4-11Al might be encoded by one or even several of these aldehyde-responsive ORs. We hence submitted Z4-11Al to an *in vitro* screening of all human ORs and their most frequent genetic variants, using heterologous expression in HEK-293 cells and a luminescence-based assay (Noe et al., 2017a, 2017b). This screening renders OR10A6 the single most responsive receptor for Z4-11Al. A subsequent olfactory panel test confirmed the results of an *in vitro* dose-response test of synthetic aldehyde analogs, showing that we discriminate between structurally related aldehydes and that our olfactory perception of Z4-11Al is remarkably sensitive and specific. The scent of the fly illustrates how chemical ecology research inspires the discovery of OR ligands and provides an account for convergent chemical communication across phylogenies.

## Results

### Sensory evaluation of fly odor

Comparative chemical analysis of volatiles released by *D*. *melanogaster* male and female flies, followed by sensory evaluation of fly odor and synthetic compound by a professional wine panel, strongly suggest that Z4-11Al is the scent of the female fly (Lebreton et al., 2017; Becher et al., 2018).

**Figure 1:**
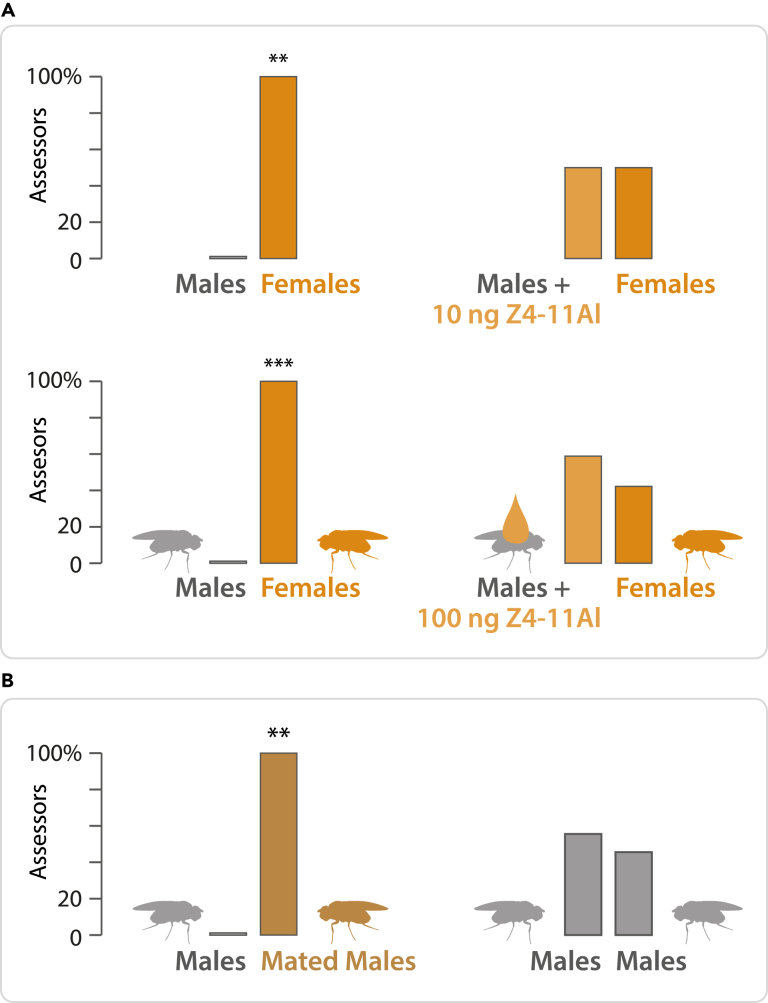
Fly painting with Z4-11Al Assessors participating in these experiments sensed 10 ng synthetic Z4-11Al formulated in water, according to a preliminary test. (A) All assessors distinguished the odor of 10 males vs 10 females emanating from glass vials, where flies had been kept during 30 min, 1 h before the onset of the experiment (*n* = 10 and 12 assessors in upper and lower panel, respectively; Chi2 = 10.0752, *p* = 0.001503 and Chi2 = 12.0, *p* = 0.005). Sex discrimination was no longer significant, when 10 and 100 ng Z4-11Al, respectively, was added to the male vials (Chi2 = 0.5952, *p* = 0.4404; Chi2 = 0.1777, *p* = 0.6733). (B) All assessors discriminated the odor of mated vs unmated males, emanating from glass vials, where flies had been kept during 30 min, 1 h before the experiment (*n* = 11 assessors; Chi2 = 10.9925, *p* = 0.0009). The control experiment with unmated males did not show differences (Chi2 = 1.0017, *p* = 0.3169).

To substantiate these findings, we compared females vs males painted with Z4-11Al. For this particular experiment, assessors were chosen according to their capacity to recognize the scent of synthetic Z4-11Al, at 10 ng formulated in water, during preliminary experiments. Assessors evaluated fly odor emanating from glass vials, which contained 10 males and 10 females, respectively, during 30 min, 1 h before the experiment. All assessors readily distinguished male and female vials. After adding 10 or 100 ng of Z4-11Al, discrimination was no longer significant (Figure 1A).

**Figure 2:**
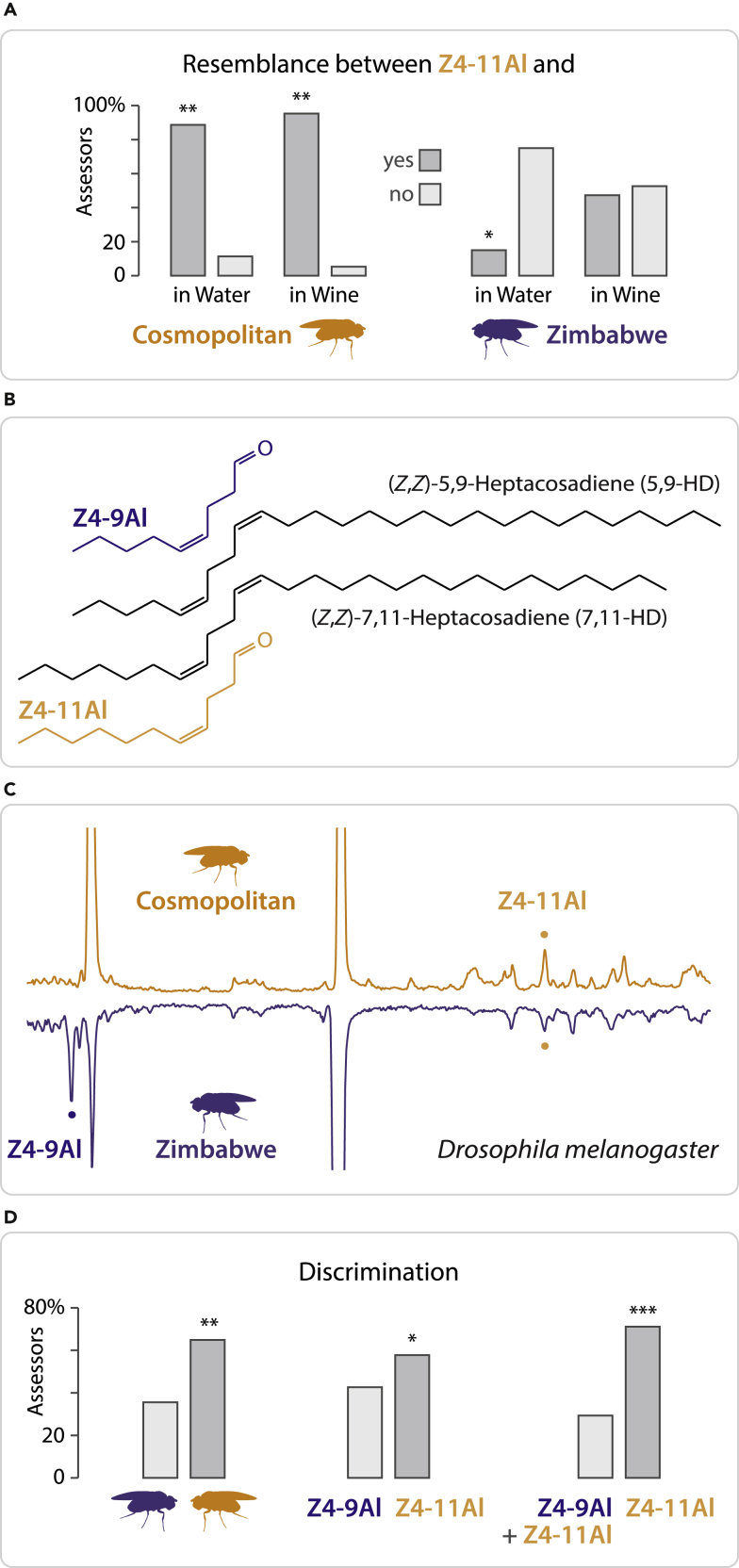
Sensory discrimination between cosmopolitan and Zimbabwe *D*. *melanogaster* females by humans, and production of Z4-11Al and Z4-9Al by these fly strains (A) Olfactory resemblance of 10 ng synthetic Z4-11Al and the odor of cosmopolitan or Zimbabwe female flies, in water and wine. Judges, who had not sensed synthetic Z4-11Al before, were asked whether or not the odors of two vials bear resemblance. Bars marked with asterisks are significantly different (*n* = 21 assessors; Chi2 = 6.9146, *p* = 0.0085; Chi2 = 10.5061, *p* = 0.0012; Chi2 = 4.9082, *p* = 0.0267; Chi2 = 0.0288, *p* = 0.8652, from left to right). (B) Z7,Z11-27Hy is the hydrocarbon precursor of the cosmopolitan *D*. *melanogaster* female pheromone Z4-11Al. Females of the Zimbabwe strain further produce Z5,Z9-27Hy and the corresponding oxidation product is Z4-9Al. (C) Chromatograms of headspace collections from batches of 60 females, with Z4-9Al and Z4-11Al highlighted. Zimbabwe flies produce a 2.6 ± 0.7-fold amount of Z4-9Al, compared with Z4-11Al (*n* = 10). (D) Olfactory discrimination of cosmopolitan vs Zimbabwe *D*. *melanogaster* females, of 10 ng Z4-9Al vs 10 ng Z4-11Al, and a 10:3-ng blend of Z4-9Al and Z4-11Al vs 10 ng Z4-11Al. Bars marked with asterisks are significantly different (*n* = 45 judges chosen at random, Chi2 = 8.7154, *p* = 0.0032; Chi2 = 5.4206, *p* = 0.0199; Chi2 = 11.4387, *p* = 0.0007, from left to right).

We further asked whether females transmit Z4-11Al or its precursor Z7,Z11-27Hy (Figure 2B) to males during mating. All assessors readily distinguished between vials impregnated with the odor of mated and unmated males, respectively (Figure 1B) and all assessors recognized the scent of Z4-11Al in vials impregnated by mated males.

We next compared synthetic Z4-11Al with the scent of *D*. *melanogaster* females, of the cosmopolitan and the Zimbabwe strains, in water and wine, providing a rich odorant background (Figure 2A). A professional wine panel was employed for this test, with no previous experience with Z4-11Al. Vials, where five fly females had been kept and released before testing, and vials formulated with 10 ng Z4-11Al, were filled with water or wine, respectively. In vials with water, panelists found the odor of Z4-11Al to resemble cosmopolitan, but not Zimbabwe flies. Even in wine, most panelists readily perceived cosmopolitan fly odor and found it to resemble synthetic Z4-11Al. In contrast, the evaluation of Zimbabwe fly odor vs Z4-11Al was impaired in wine (Figure 2A).

### Aldehyde emission by cosmopolitan and African flies

Cosmopolitan *D*. *melanogaster* females produce the courtship pheromone Z7,Z11-27Hy, which affords Z4-11Al as an oxidation product (Billeter et al., 2009; Lebreton et al., 2017). Females of the Zimbabwe strain produce mainly Z5,Z9-27Hy instead (Dallerac et al., 2000), and consequently these flies would therefore also release Z4-9Al (Figure 2B).

Headspace analysis of *D*. *melanogaster* confirmed that females of the cosmopolitan strain released Z4-11Al, whereas Z4-9Al was not detected in cosmopolitan fly effluvia collections. Zimbabwe females, on the other hand, produced Z4-9Al, in addition to Z4-11Al, at a 2.6 ± 0.7-fold amount (*n* = 10) (Figure 2C).

### OR10A6 is tuned to Z4-11Al

**Figure 3:**
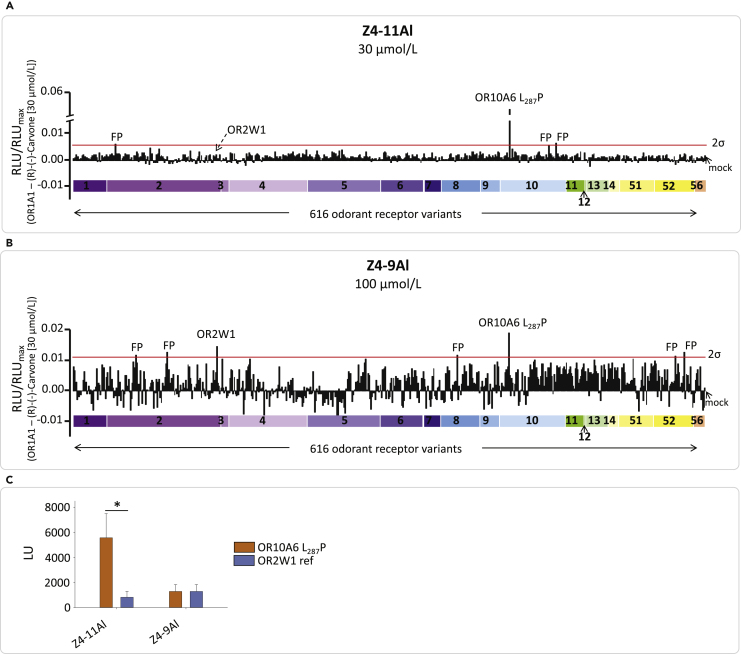
Screening of 616 recombinant human IL-6-HaloTag®-OR variants, with Z4-9Al and Z4-11Al, using an HEK-293 cell-based GloSensor™ cAMP-luminescence assay The cDNA expression plasmid OR library is shown in Table S1. (A) OR10A6 L_287_P emerges as the sole OR responding to 30 μmol/L of Z4-11Al, beyond a 2σ-threshold (red line). OR2W1 (dashed arrow) became activated only at higher concentrations (see Figures 3C and 4). (B) Both OR2W1 and OR10A6 L_287_P were activated by 100 μmol/L of Z4-9Al. Data for both screenings (relative luminescence units, RLU) were normalized to the signal amplitude of OR1A1 in response to 30 μmol/L *R*-(−)-carvone. OR families are color-coded and sorted in an ascending numerical order. The negative controls were cells transfected with a “mock” plasmid lacking any receptor coding region; false positives (FP) are indicated. (C) OR2W1 shows a significantly lower amplitude than OR10A6 L_287_P in response to Z4-11Al, but not in response to Z4-9Al. Data show mock control-subtracted raw data (luminescence units, LU) in response to 100 μmol/L of the respective aldehyde (mean ± SD, *n* = 3), the asterisk shows a significant difference (paired two-tailed *t*-test; *t* = −4.14887, *p* = 0.0142).

OR10A6 L_287_P, the only known functional OR10A6 variant (Olender et al., 2012, 2013; Trimmer and Mainland, 2017), showed by far the strongest response to 30 μmol/L of Z4-11Al beyond a 2σ-threshold, in a screening of 616 human OR variants expressed in HEK-293 cells (Figure 3A). Screening the aldehyde analog Z4-9Al at 100 μmol/L revealed two responding receptors beyond a 2σ-threshold, OR2W1 and OR10A6 L_287_P, with comparable amplitudes (Figure 3B). At 100 μmol/L Z4-11Al, OR2W1 showed an about six-fold lower response, compared with OR10A6 L_287_P (Figure 3C), despite a slightly better surface expression of OR2W1 as compared with OR10A6 L_287_P, in both HEK-293 and NxG 108CC15 cells (Figure S1).

**Figure 4:**
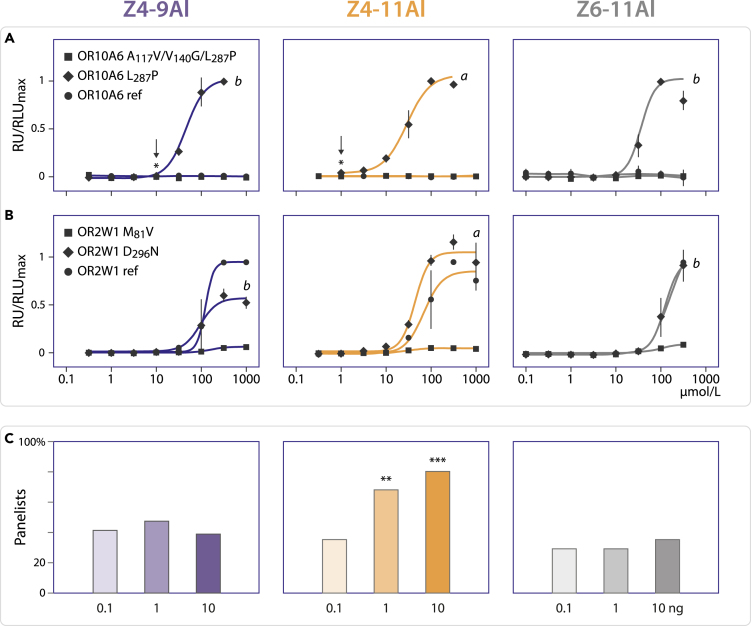
Dose-response effect of synthetic Z4-11Al, Z4-9Al, and Z6-11Al on the most frequent OR10A6 and OR2W1 haplotypes in an HEK-293 cell-based GloSensor™ cAMP-luminescence assay and an odorant panel (A) Z4-11Al is the most potent agonist for OR10A6 L_287_P, the other two OR10A6 variants were not functional (no response detected up to 1.000 μmol/L). (B) The response of OR2W1 variants was throughout weaker, compared with OR10A6 L_287_P. Relative luminescence units (RLU) were mock control-subtracted, normalized to the highest response of either OR10A6 L_287_P or OR2W1 ref, and the respective aldehyde (mean ± SD, *n* = 3). Letters show significant differences of the response of OR10A6 L_287_P (*p* = 0.0061) and OR2W1 D_296_N (*p* = 0.0048) to the test compounds, according to ANOVA followed by Friedman’s test. Responses of OR2W1 ref were not different (*p* = 0.3519). (C) A significant number of panelists (*n* = 31) detected Z4-11Al at 1 and 10 ng (Chi2 = 7.3459, *p* = 0.0067; Chi2 = 14.1607, *p* = 0.0002, respectively).

A dose-response assay further confirmed that Z4-11Al was the most potent agonist for OR10A6 L_287_P, compared with the analog Z4-9Al and the positional isomer Z6-11Al (Figure 4A). The EC_50_ values for these three aldehydes on OR2W1 haplotypes were about 2- to 3-fold higher, throughout, compared with OR10A6 L_287_P (Table 1, Figure 4B). All other ORs that responded to Z4-11Al or Z4-9Al beyond a 2σ-threshold in the screening experiments could not be validated in concentration-response assays (Figures S2, S3, and S4), suggesting <2% false positives. Among the most abundant OR10A6 haplotypes (Olender et al., 2012), OR10A6 L_287_P was functional (Figures 4A and S4).

**Table 1:** EC_50_ values for OR10A6 and OR2W1 haplotypes in response to Z4-9Al, Z4-11Al, and Z6-11Al

	Z4-9Al	Z4-11Al EC_50_ [μmol/L]^a^	Z6-11Al
OR10A6 ref	ND^b^	ND	ND
OR10A6 L_287_P	41.15 ± 14.24	28.21 ± 12.65	34.78 ± 9.21
OR10A6 A_117_V/V_140_G/L_287_P	ND	ND	ND
OR2W1 ref	119.31 ± 23.09	65.11 ± 35.75	103.73 ± 27.01
OR2W1 M_81_V	142.09 ± 92.38	ND	ND
OR2W1 D_296_N	91.82 ± 46.19	40.42 ± 11.98	137.89 ± 54.98

a Mean ± SD (*n* = 3).

b No response detected up to 1000 μmol/L.

For the ensuing panel test, 31 assessors were chosen at random. The odorant panel corroborated that we are more sensitive to Z4-11Al than to Z4-9Al or Z6-11Al (Figure 4C). A significant number of panelists sensed Z4-11Al already at 1 ng/mL in water (0.006 μmol/L). In comparison, the response to Z4-9Al or Z6-11Al was not significant, at the amounts tested.

A low response threshold to Z4-11Al *in vitro* (Figures 3 and 4) corroborates our remarkable sensitivity to the female pheromone of cosmopolitan *D*. *melanogaster*, which is only a minor compound of fly headspace (Figure 2C; Lebreton et al., 2017). Most panelists who discriminated Z4-11Al from control (Figure 4C) perceived the aroma of the pure compound to be fruity and pleasant, whereas a fly female or synthetic Z4-11Al was found to disturb wine aroma (see also Becher et al., 2018).

According to the triangle test shown in Figure 4C, 21 of 31 panelists (68%) sensed Z4-11Al at 1 ng. In a large human population, only 35% individuals carry the functional haplotype OR10A6 L_287_P (Olender et al., 2012, 2013), (Table 2) which is contradictory at first sight. We therefore genotyped 29 of the 31 panel members for OR10A6 and OR2W1. The functional variant OR10A6 L_287_P was found in 14 individuals (48.3%), and OR2W1 D_296_N was found in 26 individuals (89.7%), among the 29 panelists.

**Table 2:** OR10A6 and OR2W1 variants and their frequencies according to human genome databases (Olender et al., 2012, 2013; Howe et al., 2021)

	Accession number [1] or variant ID [2]	Minor allele frequency (%) [2]	Frequency (%) [3]
OR10A6 ref	NM_001004461.2		21.81
OR10A6 V_140_G	rs7933807	41.3	
OR10A6 A_117_V	rs7928451	39.4	0.08
OR10A6 L_287_P	rs4758258	21.2	35.02
OR10A6 A_117_V/V_140_G/L_287_P			37.33
OR10A6 V_140_G/L_287_P			1.92
OR10A6 A_117_V/L_287_P			0.15
OR2W1 ref	NM_030903.3		72.43
OR2W1 M_81_V	rs34892006	3.9	3.61
OR2W1 D_296_N	rs35771565	24.9	23.96

[1] NCBI Resource Coordinators (2017).

[2] Howe et al. (2021).

[3] Olender et al. (2012, 2013).

In a triangle test (3 vials), where 2 vials are the same, panelists are asked to pick the odd vial. The 29 anonymously genotyped panelists produced 20 correct answers (Figure 4C shows results for 31 panelists). Assuming that the 14 panelists carrying OR10A6 L_287_P picked the odd vial because they sensed Z4-11Al, and that every third of the remaining 15 panelists picks the odd vial by chance, we expect 19 correct answers, which is appreciably close to the 20 correct answers obtained with 1 ng Z4-11Al. The number of correct answers, from these 29 panelists, increased to 24 at 10 ng Z4-11Al. This might be owing to OR2W1 D_296_N, which shows a 43% higher EC50 value in response to Z4-11Al in the HEK assay, compared with OR10A6 L_287_P (Table 1).

### Discrimination between Z4-11Al and a blend of Z4-9Al and Z4-11Al

As we perceive Z4-11Al at the amounts produced by females, and not Z4-9Al, Zimbabwe and cosmopolitan flies should smell the same (Figures 2A and 2D; 4C). Females of these flies are expected to differ only with respect to odor intensity, not quality – unless a blend of Z4-11Al and Z4-9Al produces a different aroma than Z4-11Al alone. This is indeed the case. A triangle test involving 45 randomly selected panelists at SLU Alnarp shows a clear distinction between Z4-11Al and a 3:10-blend of Z4-11Al and Z4-9Al mimicking the scent of cosmopolitan and Zimbabwe flies, respectively (Figures 2C and 2D).

## Discussion

### Z4-11Al is a ligand for the highly expressed OR10A6

Human olfactory perception of Z4-11Al, the female pheromone of cosmopolitan *D*. *melanogaster* is highly sensitive and specific. An odorant panel sensed synthetic Z4-11Al at 1 ng/mL (0.006 μmol/L) and at subnanogram amounts released by single flies, and distinguished Z4-11Al from the structurally similar aldehydes Z4-9Al and Z6-14Al, or a blend of Z4-11Al and Z4-9Al. *In vitro* screening showed that the functional variant of OR10A6, which ranks among the most highly transcribed ORs in the olfactory epithelium (Saraiva et al., 2019; Verbeurgt et al., 2014), is most sensitively tuned to Z4-11Al. OR10A6 may also be tuned to other odorants, such as cyclamen aldehyde (Duroux et al., 2020), and further experiments are needed to map its entire agonist space.

OR2W1, the other receptor that showed a significant response to the *Drosophila* aldeydes in our OR screening, is a most broadly tuned human OR (Saito et al., 2009; Geithe et al., 2017a; Haag et al., 2021). A combination of highly selective and broadly tuned ORs is the basis for sensing a diverse odorant environment with only a limited number of ORs (Geithe et al., 2017a; Block, 2018; Saraiva et al., 2019; Kurian et al., 2021). Theoretical and data-based models predict that mixed OR populations of different receptive ranges enable greater odor coverage (Alkasab et al., 2002; Fonollosa et al., 2012). OR2W1 may accordingly participate together with OR10A6 in enabling receptor activity patterns in response to aldehydes, even though the transcript levels of OR2W1 in the olfactory epithelium are low, in comparison with the highly expressed OR10A6 (Verbeurgt et al., 2014; Saraiva et al., 2019). Other human ORs with an affinity to various odor-active aldehydes (Nara et al., 2011; de March et al., 2015; Block, 2018) did not respond significantly to Z4-11Al and Z4-9Al.

Remarkably, the panel reliably discriminated between Z4-11Al and a blend of Z4-11Al and Z4-9Al. That the blend afforded an entirely different hedonic quality explains how we distinguish between cosmopolitan and Zimbawe flies. Response of OR2W1 to Z4-9Al and of OR10A6 to both compounds, Z4-9Al and Z4-11Al, suggests that input from 2 ORs produces a different perception. On the other hand, as OR10A6 was responsive to both compounds, it is even possible that modulation at the OR level encodes this blend discrimination. Processing of odorant interactions is not restricted to higher olfactory circuits, but occurs even peripherally, owing to synergistic and antagonistic responses of olfactory neurons to odor mixtures. Odorant interaction and encoding of mixtures at the OR level substantially extends the receptive range of ORs (Brann and Datta, 2020; De March et al., 2020; Inagaki et al., 2020; Xu et al., 2020).

It is further intriguing that we sense the small amounts of Z4-11Al released by single flies against the rich bouquet emerging from a glass of wine. Z4-11Al is only a minor compound among the volatiles released by *Drosophila* females (this study; Lebreton et al., 2017; Becher et al., 2018), whereas the bouquet of wine is overwhelmingly complex and comprises many volatiles at far larger amounts, including a suite of aldehydes (Swiegers et al., 2005; Cullere et al., 2007).

Single ORs and their key ligands play indeed a central role in olfactory object recognition, especially against heterogeneous backgrounds. Olfactory sensory neurons expressing high-affinity ORs with low activation thresholds have been shown to become activated early during a sniff and thus accentuate the response to behaviorally salient signals, whereas input from other ORs is temporarily tuned down (Wilson et al., 2017; Arneodo et al., 2018; Bolding and Franks, 2018; Dewan et al., 2018). The odorant panel attributed a pleasant, fruity aroma to Z4-11Al as a single compound. That the admixture of Z4-11Al to wine is perceived as unpleasant may accordingly be owing to a reduced or modulated perception of wine volatiles. Similarly, wine aroma is disturbed by larger amounts of (*E*)-2-decenal, a component of a hemipteran bug defensive secretion (Mohekar et al., 2017). Conversely, a suite of unsaturated, odor-active aldehydes from coriander, including (*E*,*E*)-2,4-undecadienal, had a deodorizing effect on the malodor of porc intestines (Kohara et al., 2006; Ikeura et al., 2010).

Taken together, our observations illustrate how a key compound contributes to olfactory perception via a single OR, in addition to combinatorial coding of odorant blends by arrays of several ORs (Mainland et al., 2014). Sensitivity is, in addition to ligand affinity, a function of OR expression in olfactory sensory neurons (van der Linden et al., 2020) and OR10A6 is among the most highly expressed ORs in our nose (Verbeurgt et al., 2014; Saraiva et al., 2019).

### Occurrence of Z4-11Al in human odor scenes

Highly abundant ORs are plausibly dedicated to odorants of critical physiological, behavioral or ecological function. This raises the question of what Z4-11Al may mean to us. Perception of the same odorant by insects and vertebrates is convergent, as the respective ORs share ligand affinity, but are built differently and lack a common phylogenetic root (Su et al., 2009; Bear et al., 2016). If the convergent evolution of the vertebrate and invertebrate olfactory systems reflects an underlying logic rather than shared developmental principles (Wang et al., 2021), it would follow that convergent perception of messenger chemicals points to a behavioral role.

What is the source of Z4-11Al in a human odorscape? Animals, plants, and associated microbes each release many hundreds of compounds and these volatile emissions change with age, phenology, and physiological state (Knudsen et al., 1993; El-Sayed, 2020; Lemfack et al., 2018; Ljunggren et al., 2019). Z4-11Al has not been searched for, synthetic standards are not available commercially, and we can therefore safely assume that the occurrence of Z4-11Al is only incompletely known.

Although Z4-11Al is not considered to be a key food odorant (Dunkel et al., 2014), it has been found in coriander and clementine (Chisholm et al., 2003; Eyres et al., 2005), and similar aldehydes are typical for other fruit (e.g. Fischer et al., 2008; Chai et al., 2012). Monoenic aldehydes are perceived as “citrusy,” but also as “tallowy” as they contribute to the flavor of cooked or roast food and meat, including rice, oils, fish, chicken, and beef (Cha et al., 1992; Siegmund and Pfannhauser, 1999; Rochat and Chaintreau, 2005; Roh et al., 2006; Yang et al., 2008; Oueslati et al., 2018; Giuffre et al., 2020), and Z4-11Al has also been found in oxidized tallow (Shi et al., 2013).

Intriguingly, Z4-11Al and close analogs appear even in a pheromonal context, in several animals and humans. The crested auklet, a colonial breeding sea bird, releases a tangerine-scented, social odor that signals mate quality and contains (Z)-4-decenal as the main compound (Douglas et al., 2001; Hagelin et al., 2003). Unsaturated aldehydes are part of human scent profiles and serve as diagnostic and forensic cues (Curran et al., 2007; Li, 2014; Duffy et al., 2018; Tavares et al., 2019). Milk from humans and rabbits contains 2-nonenal and 2-undecenal, respectively (Schaal et al., 2003; Sandgruber et al., 2012), and newborn mice emit 4-nonenal (Lacalle-Bergeron et al., 2021). (*E*)-2-undecenal occurs, with several other 2-unsaturated aldehydes, in anogenital gland secretions in Pandas (Zhou et al., 2021) and Z4-11Al has been found in rabbit anal glands, accelerating heartbeat upon perception (Goodrich et al., 1978).

Taken together, Z4-11Al is found in human food, it might even be produced by ourselves and could manifest food, social context, or both. A dual function of certain pyrazines as key food odorants and semiochemicals, selectively activating the same, single human OR, has recently been demonstrated (Marcinek et al., 2021).

### Role of Z4-11Al in *D*. *melanogaster*

The vinegar fly is our involuntarily domesticated animal, since it accompanied the human global expansion out of Africa. Cosmopolitan vinegar flies are associated with us on all continents and most climate zones, they are strictly anthropophilic, depend on our food and dwellings for survival and we share a taste for fermenting food (Lachaise and Silvain, 2004; Nielsen et al., 2017; Arguello et al., 2019). *D*. *melanogaster* females, not males, produce dienic hydrocarbons that give rise to monoenic aldehydes, which is why we smell the female flies (Everaerts et al., 2010; Lebreton et al., 2017).

The sibling species *Drosophila simulans* has also attained worldwide distribution in association with humans, but is, unlike *D*. *melanogaster*, not a strict commensal and more rarely found in households or buildings (Lachaise and Silvain, 2004). *D*. *simulans* females do not produce dienic hydrocarbons, which is a main element of the mating barrier between these species. The cuticular hydrocarbon Z7,Z11-27Hy promotes courtship in cosmopolitan *D*. *melanogaster*, and suppresses interspecific matings with *D*. *simulans*, owing to differential, species-specific coding of Z7,Z11-27Hy in neural circuits mediating reproductive behavior (Billeter et al., 2009; Billeter and Wolfner, 2018; Seeholzer et al., 2018; Sato and Yamamoto, 2020).

Cosmopolitan and African *D*. *melanogaster* strains also differ with respect to cuticular hydrocarbons. The female-specific desaturase gene desat2, which affords Z5,Z9-27Hy and Z4-9Al, is functional only in African and not in cosmopolitan flies (Dallerac et al., 2000; Grillet et al., 2012). This hydrocarbon polymorphism yields a distinctive aldehyde blend, which is how we differentiate the scent of these two fly strains. Species-specific differences in hydrocarbons align with corresponding aldehyde signatures, that entail behavioral consequences. Z4-11Al attracts *D*. *melanogaster*, but not males of the Zimbabwe strain, and has an antagonistic effect on upwind flight attraction in *D*. *simulans*. This underlines the role of female-produced volatile pheromones in long-range mate communication in *Drosophila* (Lebreton et al., 2017; Borrero-Echeverry et al., 2022).

Panel tests evaluating male and female fly odor unexpectedly discovered that Z4-11Al, in addition to its hydrocarbon precursor, is among the “chemical words exchanged by *Drosophila* during courtship and mating” (Jallon, 1984). At close range, Z4-11Al stimulates courtship in males (Borrero-Echeverry et al., 2022), whereas the transfer of Z4-11Al may be a factor in reducing courtship success of freshly mated males (Scott et al., 1988). That Z4-11Al by itself is attractive to females (Lebreton et al., 2017; Borrero-Echeverry et al., 2022), even points to an antagonistic interaction with a male-produced compound, such as 11-cis-vaccenyl acetate.

### Sensory drive and convergence

Convergent perception of Z4-11Al in humans and flies could be coincident or interconnected. ORs readily adapt to habitats and to dietary or social chemosensory niches, in insects and vertebrates alike (Bear et al., 2016; Hughes et al., 2018; Saraiva et al., 2019). Transcript variants of the fly receptor DmelOR69a (Robertson et al., 2003) are tuned to food odorants and the female pheromone, respectively, and are co-expressed in the same OSNs (Lebreton et al., 2017). These twin ORs yield a degree of freedom for the acquisition of new ligands, if only they match the food and mate-finding theme.

Habitat selection and specific mate recognition are tightly interconnected (Paterson, 1985; Endler, 1992; Boughman, 2002), and the interaction between natural and sexual selection has been shown to affect cuticular hydrocarbon composition and mate recognition in *D*. *melanogaster* (Blows, 2002). We are food and home to the flies, they depend on us for survival. A commensal lifestyle is expected to generate a sensory drive and select for odorants to mediate fly aggregation and premating communication – if these odorants are produced by the flies, and if they are, in addition, characteristic elements of human odor scenes. Convergent perception of Z4-11Al is reminiscent of dedicated olfactory channels for geosmin that alert flies and humans about the presence of mold, which is detrimental for all animals (Maga, 1987; Stensmyr et al., 2012).

### Conclusion

Sensing the scent of a single fly is out of the ordinary, especially as the cue is the fly’s sex pheromone . Yet, only the discovery that a most highly expressed human OR is tuned to this pheromone underlines the biological significance of this observation. Sensitive and specific perception encourages the hypothesis that Z4-11Al is found in human habitats, where humans, domesticated animals, or shared food resources, including associated microorganisms, could be the source.

Ambient odorscapes contain countless chemicals of yet unknown activity. Our study highlights how the identification of key OR ligands leads to the discovery of messenger chemicals and delivers insights into how chemical communication interconnects species across phylogenies. Regrettably, we can barely speculate what the fly pheromone may mean to us and whether it signals food, social context, or both. Satisfying our curiosity is an excellent reason to pursue, as the vinegar fly continues to afford fundamental discoveries and studying fly sex perfumes may perhaps teach us about our own.

### Limitations of the study

The significance of Z4-11Al for humans is yet unknown. Z4-11Al and close analogs are found in in food, and mediate communication between animals. In the search for sources in human environments, close attention must be paid to occurrence of trace amounts of Z4-11Al, in view of our sensitivity. Screening for other ligands, combined with structure-activity studies, will help to elucidate the behavioral relevance of the OR10A6 channel. Last but not least, we cannot entirely exclude that yet other ORs or OR variants may participate in the perception of Z4-11Al.

## STAR★Methods

### Key resources table

**Table:** 

REAGENT or RESOURCE	SOURCE	IDENTIFIER
**Bacterial and virus strains**		

XL1-Blue Competent Cells	Agilent Technologies, Inc.	CAT#200236

**Chemicals, peptides, and recombinant proteins**		

(*Z*)-4-undecenal	Erika A. Wallin, Mid Sweden University	N/A
(*Z*)-6-undecenal	Erika A. Wallin, Mid Sweden University	N/A
(*Z*)-4-nonenal	Erika A. Wallin, Mid Sweden University	N/A
D-luciferin (beetle) monosodium salt	Promega	CAT#E464X

**Deposited data**		

Odorant Receptor Screening, Chemical Analysis, Panel tests	Mendeley Data, https://doi.org/10.17632/dkpxj9ckkv.1	N/A

**Experimental models: Cell lines**		

Human: HEK293 cells	ATCC	CRL-1573

**Experimental models: Organisms/Strains**		

Human adults: sensory panels	Systembolaget, Stockholm; Dept Plant Protection Biology, Swedish University of Agricultural Sciences, Alnarp	N/A
*Drosophila melanogaster* Dalby	SLU Alnarp	N/A
*Drosophila melanogaster* Zimbabwe	Bloomington	RRID:BDSC_60741

**Oligonucleotides**		

Primers for molecular cloning of human ORs, see Table S2	This paper	N/A
Primers – vector internal, see Table S3	This paper	N/A
Primers for site-directed mutagenesis, see Table S4	This paper	N/A
Primers for haplotype sequencing, see Table S5	This paper	N/A

**Recombinant DNA**		

Plasmid: pFN210A	Promega	CAT#pFN210A SS-HaloTag® CMV-neo Flexi®- Vector
Plasmid: pGloSensor^TM^-22F	Promega	CAT#E2301
Plasmid: RTP1S	Saito et al. (2004)	N/A
Plasmid: Gαolf	Shirokova et al. (2005)	N/A
Plasmid: Gγ13	Li et al. (2013)	N/A

**Software and algorithms**		

SigmaPlot 14.0	Systat Software	N/A
Prism 9.3	GraphPad	N/A

### Resource availability

#### Lead contact

Further information and requests for resources should be directed to and will be fulfilled by the Lead Contact, Peter Witzgall (peter.witzgall@slu.se).

#### Materials availability

This study did not generate new unique materials or reagents.

### Experimental model and subject details

#### Insects

Cosmopolitan (Dalby) and Zimbabwe (S-29, Bloomington) strains of *D*. *melanogaster* were reared on a standard sugar-yeast-cornmeal diet at room temperature (25 ± 2°C) and 50 ± 5 rH under a 12:12-h L:D photoperiod. When preparing experiments, eclosing flies were collected every 4 h and sexed, according to the sex comb on the third segment of the male forelegs. Flies were tested when 3 days old. Presence of meconium was used as a distinguishing feature for virgin flies. Females and males were kept separately in 30-mL Plexiglas vials with fresh food.

#### Sensory panel

The Swedish Alcohol Retailing Monopoly (Systembolaget) continuously monitors product quality. A sensory panel of professional assessors at Systembolaget consisted of 9 women and 12 men, with an average age of 41.8 ± 10.9 y. At SLU Alnarp, panel members were recruited from personnel at the Department of Plant Protection Biology and the Department of Biosystems and Technology, 18 women and 27 men, with an average age of 38.4 ± 14.3 years.

Panel members were informed about experimental hypotheses and protocols, the scope of the study, and potential risks. Results cannot be traced to individual persons and the study is therefore exempt from ethical review. Informed consent was obtained from all subjects and the study was approved by a local ethics committee and the Legal Affairs Unit at the Swedish University of Agricultural Sciences.

### Method details

#### Chemicals

Isomeric purity of (*Z*)-4-undecenal (Z4-11Al) was 98.6%, according to gas chromatography coupled to mass spectrometry (6890 GC and 5975 MS, Agilent Technologies, Santa Clara, CA, USA). Isomeric purity of (*Z*)-4-nonenal (Z4-9Al) and (*Z*)-6-undecenal (Z6-11Al) were 97.4 and 96%, repectively. Chemical purity of these synthetic aldehydes was >99.9%. Ethanol (redistilled; Merck, Darmstadt, Germany) was used as solvent.

For the OR screening assays, the following chemicals were used: Dulbecco’s MEM medium (#F0435), FBS superior (#S0615), L-glutamine (#K0282), penicillin (10000 U/ml)/streptomycin (10000 μg/mL) (#A2212), trypsin/EDTA solution (#L2143) (Biochrom, Berlin, Germany), CaCl2∗2H2O (#22322.295), D-glucose (#101174Y), dimethyl sulfoxide (DMSO) (#83673.230), HEPES (#441476L), potassium chloride (#26764.230), and sodium hydroxide (#28244.295) (VWR Chemicals BDH Prolabo, Leuven, Belgium), sodium chloride (#1064041000, Merck, Darmstadt, Germany), ViaFect™ Transfection Reagent (#E4981, Promega, Walldorf, Germany), D-luciferin (beetle) monosodium salt (#E464X, Promega, Walldorf, Germany), Pluronic® PE 10500 (#500053867, BASF, Ludwigshafen, Germany), (R)-(−)-carvone (#W224908, Sigma-Aldrich, Steinheim, Germany).

#### Pheromone collection and chemical analysis

Sixty unmated 3-d-old cosmopolitan and Zimbabwe females (n = 9 and n = 10, respectively) were transferred to standard glass rearing vials (24.5 × 95 mm, borosilicate glass; Fisher Scientific, Sweden), which had been baked at 350°C overnight. After 24 h, the flies were removed and the vial was rinsed with 200 μL of hexane, containing 100 ng decanal as internal standard, in an ultrasonic water bath for 3 min. The solvent was transferred to 1.5 mL GC-MS vials with insert and condensed to ca. 5 μL in a fume hood.

Two μL of the solvent rinses were analyzed by gas chromatography-mass spectrometry (GC-MS) (6890 GC and 5975 MS, Agilent, Santa Clara, CA, USA) on a fused silica capillary column (60 m × 0.25 mm), coated with HP-5MS UI (d_f_ = 0.25 μm; Agilent). Injections were made in splitless mode (30 s), at 275°C injector temperature. The GC oven was programmed from 50 to 250 °C at 8 °C/min (2 and 10 min hold, respectively) and a final temperature of 275°C, the mobile phase was helium (34 cm/s). The MS operated in scanning mode. Aldehydes were identified by direct comparison of mass spectra and retention data with synthetic standards.

#### OR expression and screening

##### Cell culture and transient DNA transfection

Human embryonic kidney (HEK-293) cells were cultivated in Dulbecco’s MEM medium (DMEM: w 3.7 g/L NaHCO3, w 4.5 g/L D-glucose, w/o L-glutamine, w/o Na-pyruvate) supplemented with 10% fetal bovine serum (FBS superior), 2 mM L-glutamine, 100 units/mL penicillin, and 100 μg/mL streptomycin in 10 cm cell culture dishes at 37°C, 5% CO2, and 100% humidity, as test cell systems for the functional expression of recombinant ORs (Geithe et al., 2015, 2017b; Noe et al., 2017a). One day before transfection, HEK-293 cells were transferred with a density of 12000 cells per well in white 96-well plates (Thermo Scientific™ Nunc™ F96 MicroWell™, white, #136102, Thermo Fisher Scientific, Waltham, USA). The transfection was done by the cationic lipid-transfection method using 100 ng OR plasmid-DNA, 50 ng olfactory G-protein Gαolf (Jones and Reed, 1989; Shirokova et al., 2005), 50 ng RTP1S (Saito et al., 2004), 50 ng Gγ13 (Li et al., 2013), and 50 ng genetically modified cAMP-luciferase pGloSensor™-22F (Binkowski et al., 2009) (Promega, Madison, USA), each with ViaFect™ Transfection Reagent. As negative control, transfection of an empty pFN210A-vector-plasmid (mock) was employed. As positive control OR1A1 was transfected on each plate. For concentration-response relations, each transfection was done in triplicate on the same 96-well plate. For receptor screening experiments, all 391 human OR wild-types plus 225 of their most frequent haplotypes (altogether 616 OR variants) were transfected in duplicates. The entire OR library, including official gene symbols, haplotypes, and sequence accession numbers is given in Table S1. The cells were taken into experiment 42 h post-transfection (Geithe et al., 2015, 2017b; Noe et al., 2017a).

##### cAMP luminescence assay

Cell culture media of the transfected HEK-293 cells in the 96-well plates was replaced 1 h prior to the luminescence measurement with physiological salt solution containing 140 mmol/L NaCl, 10 mmol/L HEPES, 5 mmol/L KCl, 1 mmol/L CaCl2, 10 mmol/L D-glucose and 2% D-luciferin, pH 7.4. After this incubation, basal luminescence signals for each well (three consecutive data points, 60 s intervals) were recorded with the GloMax® Discover Microplate Reader (Promega, Madison, USA) before odorant application. As positive control, 30 μmol/L (R)-(−)-carvone was applied on the OR1A1 transfected cells. Odorant stock solutions were prepared in DMSO, and diluted 1:1000 into the physiological salt solution containing 0.05% Pluronic® PE 10500, as solvent mediator. Final DMSO concentration on the cells was 0.1%. Four min after odorants were applied to the cells, three consecutive data points at 60 s intervals were recorded for each well with the GloMax® Discover Microplate Reader.

##### Data analysis of cAMP luminescence measurements

OR library screenings. The raw luminescence data obtained from Spark® multimode microplate reader was transferred to Excel. Data points of basal level and data points after odorant application were each averaged. From each luminescence signal, the corresponding basal level was subtracted and afterwards normalized to the amplitude of the reference odorant-receptor pair (OR1A1 vs. 30μM R-(−)-carvone) on each 96-well plate. The normalized values for each receptor measured in duplicates were averaged and plotted alongside with the signal derived from mock-transfected cells. Signals above a 2σ-threshold (average of all signals plus 2-times Standard Deviation) were considered as positive hits and objected to further analysis, such as concentration-response relations. False positives were defined as signals ≥ 2σ, which did not show a concentration-dependent activation in subsequent experiments.

Concentration-response relations. The raw luminescence data obtained from the GloMax® Discover Microplate Reader were processed as followed. For each well, the average of the three data points before odorant addition was subtracted from the average of the three data points after stimulation (Δsignal). Then, the corresponding mock of each substance/concentration was subtracted from each Δsignal value. All mock-subtracted Δsignal-values were then normalized to the positive control of each plate, or to the respective maximum signal (max) of each concentration-response relation. EC_50_ values were obtained by fitting the function f(x)=((min-max)/(1+(x/EC_50_)^Hillslope^)+max) to the data.

##### Flow cytometry

Cell surface expression of OR10A6 and a selected number of other ORs, and non-olfactory GPCR, was investigated in HEK-293 cells, as well as in the neuronal cell line Neuroblastoma x Glioma (NxG) hybrid cells (Noe et al., 2017a). Cells were cultivated in 12-well plates with a density of 96,000 or 80,000 cells per well, for HEK-293 or NxG cells, respectively. On the next day the transfection was performed as described above. Cells were washed twice with serum free medium prior to FACS analyses (MACSQuant Analyzer, Miltenyi Biotec, Bergisch Gladbach, Germany). To quantify cell surface expression of recombinant ORs, cells were harvested 42 h post transfection and stained with the cell-impermeant HaloTag® Alexa Fluor® 488 Ligand (ex/em = 499/518 nm) for 30 min at 37°C in a cell culture incubator with humidified atmosphere, and 5% or 7% CO2 for HEK-293 or NxG cells, respectively. A cell line-specific forward- and side-scatter gate was set to exclude dead cells, and a fluorescence detection channel was defined. For HEK-293 cells, the forward- and side-scatter channels were set to 240 and 395V, respectively. The Alexa Fluor® 488 signal (FITC or B1-channel) was detected with 195V. For NxG cells, the forward- and side-scatter channels were set to 235 and 360V, respectively. The Alexa Fluor® 488 signal (FITC or B1-channel) was detected with 175V. In each case, 10,000 cells were measured. The analysis was performed with the FlowLogic™ analysis software (inivai Technologies, Mentone, Victoria, Australia). The gating of the AlexaFluor® 488 signal of each mock control defined the distinction between negative and positive cells. Membrane expression of receptors was determined in at least three independent transfection experiments (Figure S1).

#### Molecular cloning of human OR10A6

The protein-coding region of human OR10A6 and OR2W1 (for accession numbers see Table 2) was amplified from human genomic DNA by polymerase chain reaction (PCR), using gene-specific primers (Table S2), ligated with T4-DNA ligase (#M1804, Promega, Madison, USA) either MfeI/NotI (#R3589S/ # R0189S, New England Biolabs, Ipswich, UK) or EcoRI/NotI (#R6017/ #R6435, Promega, Madison, USA) into the expression plasmid (#pFN210A SS-HaloTag® CMV-neo Flexi®-Vector, Promega, Madison, USA), and verified by Sanger sequencing (Eurofins Genomics, Ebersberg, Germany) using vector internal primers (Table S3).

#### PCR-based site-directed mutagenesis

We generated variants of OR10A6 and OR2W1 (Table 2) by two-step PCR-based site-directed mutagenesis (Noe et al., 2017c) using gene-specific primers and overlapping mutation primers, carrying the changed nucleotides (Table S4). Final amplicons were then sub-cloned as described above, and verified by Sanger sequencing (Eurofins Genomics, Ebersberg, Germany) using vector internal primers (Table S3).

#### Sensory evaluation

Sensory panels are described above, they were of balanced sex ratio and even age distribution. Judges worked in separate fume hoods, they were given 15 min for each test. They were asked to fill protocol sheets that listed vial numbers or experimental questions with tick boxes, a scale for hedonic quality, intensity, familiarity and edibility for the respective odour stimuli, and a free text field. Judges were asked to not consume beverages or food during 1 h before the test, and to not wear perfume.

Synthetic chemicals were diluted in redistilled ethanol (Sigma-Aldrich). One h prior to testing, 10-μL aliquots were pipetted into 20-mL screw-top glass vials (Genetec) containing 2 mL redistilled water, or 2 mL white wine (Ruppertsberger Riesling, Systembolaget, 72038-01). Control vials contained 2 mL redistilled water and 10 μL ethanol. Vials were used between 1 and 2 h following formulation.

For evaluation of fly strain odour, 5 live *D*. *melanogaster* females, of the cosmopolitan (Dalby) and Zimbabwe (S-29, Bloomington) strains, respectively, were placed during 3 h in 20-mL vials, they were released ca 30 min before testing, and 2 mL of redistilled water or wine was added to the vial. A pairwise comparison comprised vials containing either 10 ng synthetic Z4-11Al or fly odour, in 2 mL water or wine. Panelists (n = 21), members of a professional wine panel at Systembolaget (Stockholm), were asked whether or not the odours in the vials bear resemblance to each other.

For comparison of cosmopolitan and Zimbabwe *D*. *melanogaster* females with Z4-9Al and Z4-11Al in a triangle test, batches of 5 flies were kept during 3 h in 20-mL vials, and released ca 30 min before testing, and 2 mL redistilled water was added to the vial (n = 45 judges; SLU Alnarp). Tests with synthetic compound comprised 10 ng Z4-9Al or 10 ng Z4-11Al alone, and a blend of 10 ng Z4-9Al and 3 ng Z4-11Al and employed the same panel (n = 45 judges). Further triangle tests evaluated increasing amounts, 0.1, 1 and 10 ng of synthetic Z4-9Al, Z4-11Al and Z6-11Al, respectively (n = 31 judges; SLU Alnarp). Of three stimuli in each triangle, two were the same and judges were asked to point out the odd sample.

For comparison of cosmopolitan (Dalby) males painted with Z4-11Al vs females, and unmated vs mated males, batches of 10 flies were kept in empty glass vials during 30 min, they were released 1 h prior to the experiment and 2 mL water was added to the vials. Judges (n = 10 to 12) at SLU Alnarp, who were each given a triangle of 2 male and 1 females vials, were first asked to identify the female vial. After adding 10 ng or 100 ng Z4-11Al, in 10 μL ethanol, to the male vials, and 10 μL ethanol to the female vial, judges were again asked to identify the female vial. For comparison of mated vs unmated males, only water was added to the vials. For the painting experiments, judges were selected according to their capacity to sense 10 ng Z4-11Al, according to preliminary experiments.

#### OR10A6 and OR2W1 genotyping in panelists

Spit samples were collected from 29 anonymized judges evaluating the dose response test with synthetic Z4-9Al, Z4-11Al and Z6-11Al. Genomic DNA samples were collected (ORAgene Dx, DNA GENOTEK) and purified (prepIT-L2P, DNA GENOTEK) from 500 μL of saliva, following the manufacturer’s instructions. The following primers were used in PCR (Q5 High-Fidelity DNA polymerase, New England Biolabs) to amplify an 850 base-pair OR10A6 fragment covering all polymorphic sites (OR10A6f: 5-TATGCCTGAAATGCTGGTGG-3’; OR10A6r: 5’-ACAATCAAACTTGGAGAACACA-3’) and a 977 base-pair OR2W1 fragment covering all polymorphic sites (OR2W1f: 5’- CTGTCAGGAGTTGTCGCCAT-3’; OR2W1r: 5’- TGGATCTCCATGACCTAGGAA-3’). Both amplicons were produced using the following cycling conditions (initial denaturation at 98°C for 30 s, 35 cycles at 98°C for 10 s, 58°C for 20 s and 72°C for 30 s, and final extension at 72°C for 2 min), with each reaction including 1 μL of purified genomic DNA and 1 μL of each primer (at 10 μM) in a final volume of 25 μL. The PCR products were purified (QIAquick PCR Purification Kit, Qiagen) and sequenced (Eurofins Genomics, Germany). The sequence chromatograms were manually analyzed for determining individual OR10A6 and OR2W1 haplotypes.

#### Ethics statement

Participants were informed about the aim of the study, potential risks, the experimental protocol and they all provided formal consent. The study was conducted in accordance with the ethical principles for research involving human subjects developed in the Declaration of Helsinki (WMA). The study is exempt from ethical review, according to the Legal Affairs Unit at the Swedish University of Agricultural Sciences (SLU.ua.2022.2.2–3234).

### Quantification and statistical analysis

Statistical tests for significance were calculated with a paired, two-tailed t-test and a Chi2-test, using Prism 9.3 (GraphPad) and ANOVA followed by Friedman's test (SAS). Statistical significance was defined as a p-value <0.05. p-values and sample sizes are given in the results section or the figure legends.

## Data Availability

• Data generated by this study are available at Mendeley (https://doi.org/10.17632/dkpxj9ckkv.1). • This study did not generate code. • Additional information required to reanalyze the data reported in this paper is available from the lead contact upon request.

## Supplemental information

Document S1. Figures S1–S4 and Tables S2–S5

Table S1. cDNA expression plasmid OR library, related to Figures 3 and 4 Table S1 is found online at Mendeley (https://doi.org/10.17632/dkpxj9ckkv.1).
