# Supplementary material for: The human odorant receptor OR10A6 is tuned to the pheromone of the commensal fruit fly Drosophila melanogaster
Source: iScience. 2022 Oct 3;25(11):105269. doi: 10.1016/j.isci.2022.105269 (PMC9589189; doi:10.1016/j.isci.2022.105269)
Supplement: Document S1. Figures S1–S4 and Tables S2–S5 [file mmc1.pdf]

iScience, Volume *25*

Supplemental information

The human odorant receptor OR10A6 is tuned to the pheromone of the commensal fruit fly *Drosophila melanogaster*

Tim Frey, Charles A. Kwadha, Franziska Haag, Julien Pelletier, Erika A. Wallin, Elsa Holgersson, Erik Hedenström, Björn Bohman, Marie Bengtsson, Paul G. Becher, Dietmar Krautwurst, and Peter Witzgall

Table S2: Oligonucleotides for molecular cloning of odorant receptors, related to Figures 3, 4.
Table S3: Vector internal oligonucleotides, related to Figures 3, 4.
Table S4: Oligonucleotides for site directed mutagenesis, related to Figures 3, 4.
Table S5. Oligonucleotides for haplotype sequencing, related to Figure 4.

Figure S1: Cell-surface expression of exemplary ORs representative of each family of ORs, related to Figures 3, 4.
Figure S2: Validation of OR hits of the OR-library screening with (Z)-4-undecenal, related to Figures 3, 4.
Figure S3: Validation of OR hits of the OR-library screening with (Z)-4-nonenal, related to Figures 3, 4.
Figure S4: OR10A6 haplotypes do not respond to the pheromone (Z)-4-undecenal and its oxidation product (Z)-4-nonenal, related to Figures 3, 4.

Table S1. cDNA expression plasmid OR library - see Mendeley (doi:10.17632/dkpxj9ckkv.1)

Table S2. Oligonucleotides for molecular cloning of odorant receptors

Gene	Oligo-nucleotide	Restriction Site	TM (°C)		Sequence 5´→3´
OR10A6	al-555	MfeI	63	fw	CTGC *CAATTG* **ATG** GAA AGA CAA AAT CAA AGC TGT GTG GTT GAA TTC ATC C
	al-556	NotI	63	rv	GCTG *GCGGCCGC* GCA GTG ACT AAA TCT TAC ATG GCT TCT CAA CAC AG
OR2W1	al-185	EcoRI	58	fw	GTGA *GAATTC* **ATG** GAC CAA AGC AAT TAT AGT TCT TTA CAT GG
	al-186	NotI	58	rv	CTGC *GCGGCCGC* ATT AGA AAA CAT ATT TAA TTG CCT TAT TCA CTC TAG TC

TM = melting temperature, fw = forward, rv = reverse. Italic letters highlight the restriction sites. Start and Stop codons are printed bold.

Table S3. Vector internal oligonucleotides

Vector	Oligonucleotide	TM (°C)		Sequence 5´→3´
pFN210A	520	60	fw	GTG GAC ATC GGC CCG GGT C
	550	52	rv	CAC AAA TAA AGC ATT TTT TTC ACT GC

TM = melting temperature, fw = forward, rv = reverse

Table S4. Oligonucleotides for site directed mutagenesis

Gene	Oligonucleotide	TM (°C)		Sequence 5´→3´
OR10A6 $P_{287}L$	tf-014	60	fw	CCA CTG CTG AAT CTG CTT ATC TAC AGT TTG
	tf-013	60	rv	GTA GAT AAG CAG ATT CAG CAG TGG TGT C
OR10A6 $V_{140}G$	tf-032	58	fw	GGA GTT TTT ATG AAA TTA ATT ATA TTT TCA TGG GCC
	tf-031	57	rv	GAA AAT ATA ATT AAT TTC ATA AAA CCT CCT TTA TTC ATA ATC
OR10A6 $A_{117}V$	tf-034	68	fw	CTT CTG GGA GGA ATG GCT TAT GAC CGA TTT GCT GCA
	tf-033	63	rv	TCG GTC ATA AGC CAT TAC TCC CAG AAG AAA ACA TTC
OR2W1 $M_{81}V$	FN-075	61	fw	CAT CAT CCC TCA GAT GCT GGT CAA CTT G
	FN-076	61	rv	CAC AAG TTG ACC AGC ATC TGA GGG ATG
OR2W1 $D_{296}N$	FN-199	59	fw	CAC CTT AAG AAA TAA GAA CAT GAA GGA TGC C
	FN-200	59	rv	CAG GGC ATC CTT CAT GTT CTT ATT TCT TAA G

TM = melting temperature, fw = forward, rv = reverse

Table S5. Oligonucleotides for haplotype sequencing

Gene	Oligonucleotide	TM (°C)		Sequence 5´→3´

OR10A6	jp10A6f	58	fw	TAT GCC TGA AAT GCT GGT GG
	jp10A6r	56	rv	ACA ATC AAA CTT GGA GAA CAC A
OR2W1	jp2W1f	60	fw	CTG TCA GGA GTT GTC GCC AT
	jp2W1r	59	rv	TGG ATC TCC ATG ACC TAG GAA

TM = melting temperature, fw = forward, rv = reverse

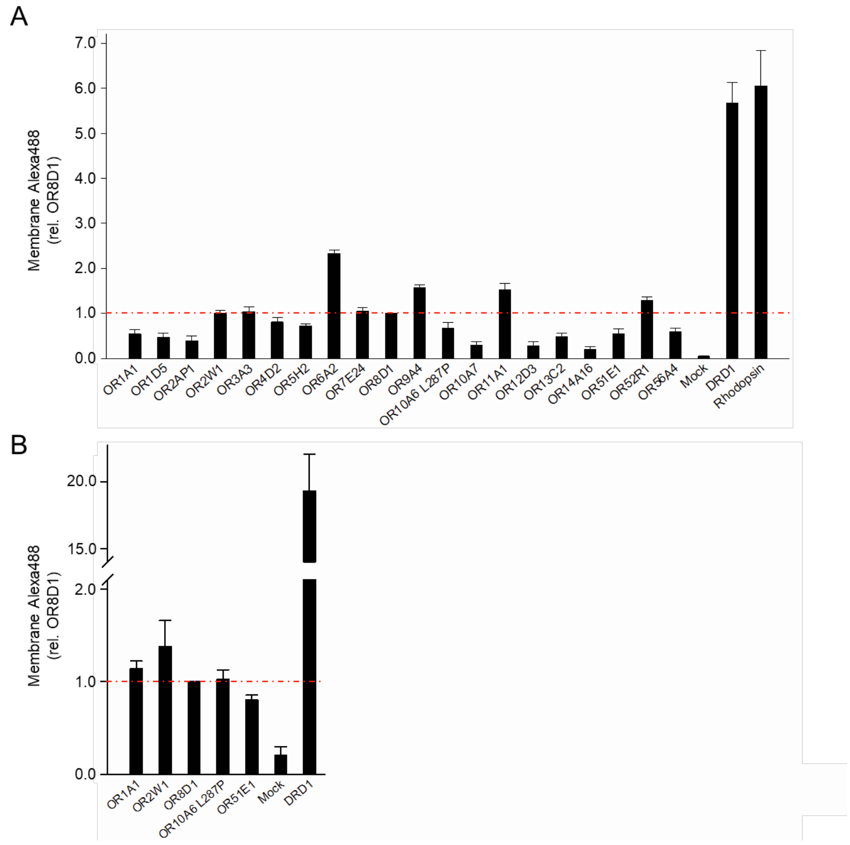

Figure S1. Cell-surface expression of exemplary ORs representative of each family of ORs

(A) Bar chart showing the relative surface expression of exemplary ORs representative of each family of ORs as well as OR10A6 $L_{287}P$ as well as the GPCRs DRD1 and Rhodopsin, using the flow cytometry assay with NxG 108CC15 cells. (B) Bar chart showing the relative surface expression of exemplary ORs as well as OR10A6 $L_{287}P$ as well as the GPCR DRD1, using the flow cytometry assay with HEK-293 cells. Data is displayed as mean ± SD (n = 3 – 7). FITC signals are significantly different from mock at $p < 0.05$ (two-tailed t-test).

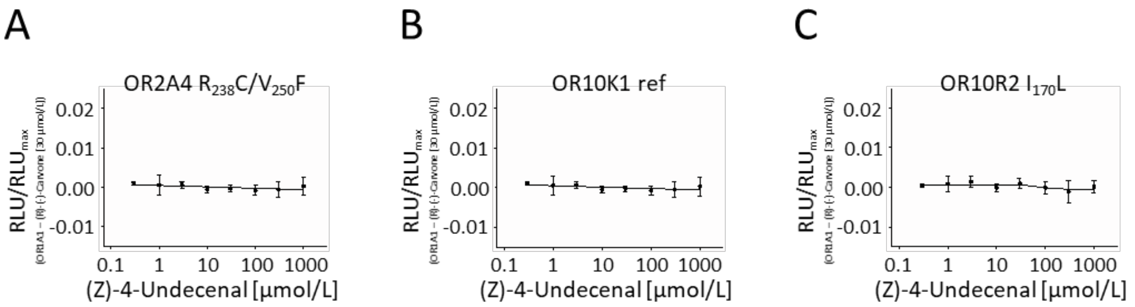

Figure S2: Validation of OR hits of the OR-library screening with (Z)-4-undecenal

Concentration-response relations of (Z)-4-undecenal on false positive OR hits of the OR-library screening (see Figure 3A). Data were mock control-subtracted, normalized to the response of OR1A1 ref to (R)-(−)-carvone (30 µmol/L), and displayed as mean ± SD (n = 3). RLU = relative luminescence unit.

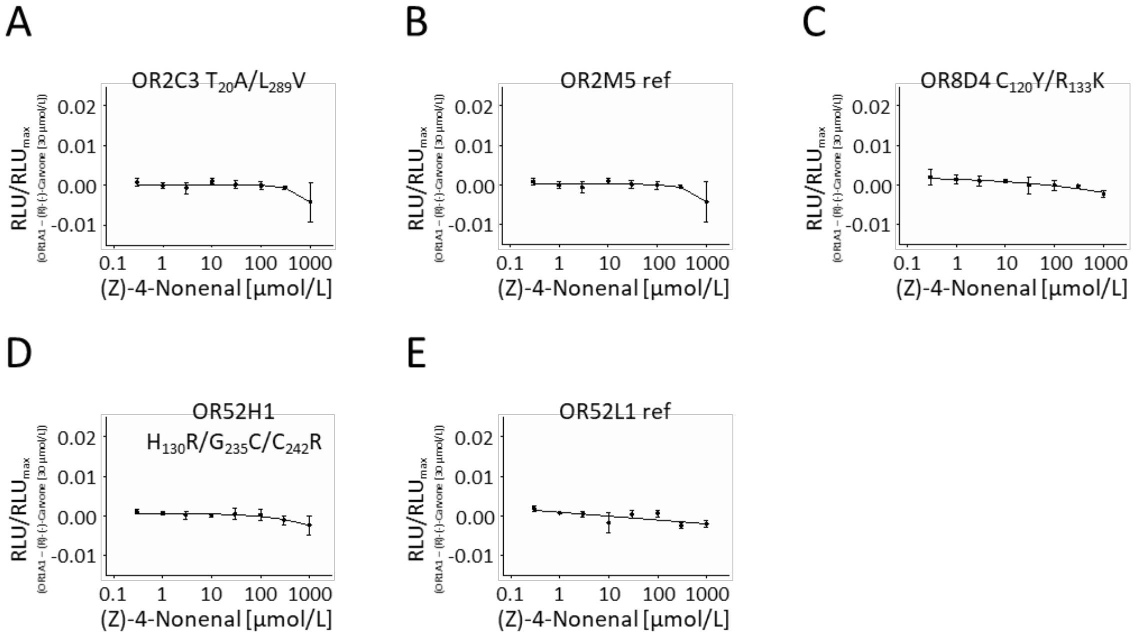

Figure S3: Validation of OR hits of the OR-library screening with (Z)-4-nonenal

Concentration-response relations of (Z)-4-nonenal on false positive OR hits of the OR-library screening (see Figure 3B). Data were mock control-subtracted, normalized to the response of OR1A1 ref to (R)-(−)-carvone (30 µmol/L), and displayed as mean ± SD (n = 3). RLU = relative luminescence unit.

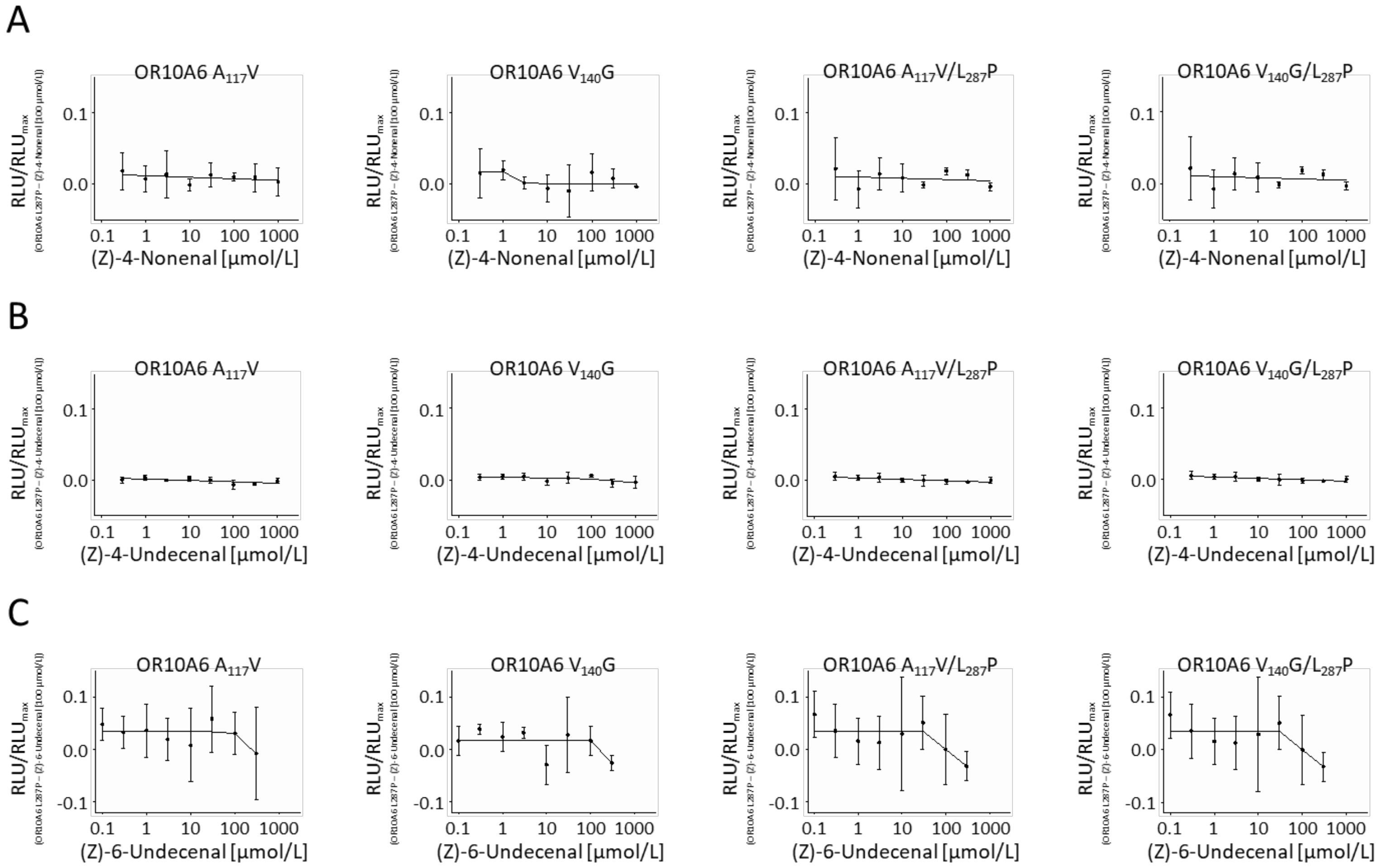

Figure S4: OR10A6 haplotypes do not respond to the pheromone (Z)-4-undecenal and its oxidation product (Z)-4-nonenal

Concentration-response relations of (Z)-4-nonenal (A), (Z)-4-undecenal (B), and (Z)-6-undecenal (C) on the OR10A6 haplotypes OR10A6 A_{117}V, OR10A6 V_{140}G, OR10A6 A_{117}V/L_{287}P, and OR10A6 V_{140}G/L_{287}P. Data were mock control-subtracted, normalized to the response of OR10A6 L_{287}P with 100 µmol/L of the corresponding aldehyde, and displayed as mean ± SD (n = 3). RLU = relative luminescence unit
